# Ruxolitinib in Patients With Chronic Active Epstein-Barr Virus Infection: A Retrospective, Single-Center Study

**DOI:** 10.3389/fphar.2021.710400

**Published:** 2021-09-06

**Authors:** Yue Song, Jingshi Wang, Yini Wang, Zhao Wang

**Affiliations:** Department of Hematology, Beijing Friendship Hospital, Capital Medical University, Beijing, China

**Keywords:** chronic active epstein-barr virus, ruxolitinib, treatment, hemophagocytic lymphohistiocytosis, inflammation, cytokines, JAK-STAT signaling pathway, ruxolitinib

## Abstract

**Background:** Chronic active Epstein-Barr virus (CAEBV) infection is one of the EBV-positive T- or NK-cell lymphoproliferative diseases. There is no safe and effective treatment currently and the only proven curable therapy is allogeneic hematopoietic stem cell transplantation (allo-HSCT). The JAK1/2 inhibitor, ruxolitinib, is now considered a novel therapy in inflammatory disease, and hypercytokinemia is an important feature of CAEBV.

**Method:** All patients who suffered active CAEBV and were treated with ruxolitinib as compassionate use in our center from Sep 1, 2017, and Apr 30, 2019, were retrospectively analyzed.

**Results:** In general, seven out of nine patients responded to ruxolitinib. Six out of seven patients became afebrile within 48 h. The AST/ALT level of three out of four patients decreased after ruxolitinib treatment. Two patients with cytopenia recovered. No significant decrease in the EBV-DNA copy number was observed (*p* = 0.161). For those seven patients who responded to ruxolitinib, the median continuing period in remission was 7.1 weeks (range, 3.4–101.0 weeks). Two patients achieved long-term stable remission with ruxolitinib monotherapy. None of these patients discontinued ruxolitinib due to the possible toxicity.

**Conclusion:** Ruxolitinib is an effective and rather safe option for controlling the inflammatory symptoms of active CAEBV, especially in patients with CAEBV who have failed previous treatments or have relapsed. It can also play a promising role in improving the quality of daily life of patients and successfully bridging to allo-HSCT.

## Introduction

Approximately 95% of adults are infected with Epstein-Barr virus (EBV). Primary EBV infection is usually asymptomatic but sometimes progresses to infectious mononucleosis, which resolves spontaneously after the emergence of EBV-specific immunity. Rare populations infected with EBV develop a life-threatening condition termed chronic active Epstein-Barr virus (CAEBV) disease (Kimura et al., 2001; Okano et al., 2005). CAEBV infection was first reported in 1986 (Olson et al., 1986). In most cases of CAEBV reported in Asians or Native Americans, EBV was detected in T- or natural killer- (NK-) cells, while in western countries, EBV infected B-cells more (Quintanilla-Martinez et al., 2000; Kimura et al., 2001). CAEBV is now regarded as a prototype of EBV-associated T-or NK-cell lymphoproliferative diseases (EBV + T/NK-LPDs) (Quintanilla-Martinez et al., 2017). In certain circumstances, CAEBV will develop to hemophagocytic lymphohistiocytosis (HLH), lymphoma, or multiorgan failure, which may lead to rapid progression and finally death (Kimura et al., 2001; Kimura et al., 2012).

The three main symptoms of CAEBV are fever, lymph node swelling, and hepatosplenomegaly/liver damage, which are “IM-like symptoms” (Sawada et al., 2017). These patients often have some of the complications found in otherwise-healthy patients with acute EBV infection, but unlike healthy patients, these complications persist and progress. Even though in most conditions, the symptoms of CAEBV patients seem to be mild and self-limited, the only proven effective therapy to cure CAEBV is allogeneic hematopoietic stem cell transplantation (allo-HSCT) (Cohen et al., 2011; Kimura et al., 2012; Sawada et al., 2017). CAEBV is invariably fatal without transplantation. Antiviral therapy is ineffective. Corticosteroids or other immunosuppressive agents often relieve symptoms, but over time patients become refractory to therapy, develop progressive immunodeficiency, and usually succumb to opportunistic infections or lymphoproliferative disease (Cohen et al., 2011; Bollard and Cohen, 2018). In 2017, Akihisa Sawada et al. (Sawada et al., 2017) proposed a “3-step strategy” for CAEBV, which is Step 1 (cooling): immunochemotherapy, Step 2 (cytoreduction): multidrug chemotherapy, and Step 3 (reconstruction): allogeneic HSCT. This therapy can effectively reduce the disease burden of patients before allo-HSCT. However, with the long-term usage of corticosteroids or cytotoxic drugs, complications such as opportunistic infections may occur, thus delaying follow-up treatment. There are also some patients who cannot tolerate the radical therapy due to organ dysfunction.

JAK-STAT pathway is the final common pathway for most inflammatory reactions. The JAK1/2 inhibitor, ruxolitinib, is now considered a novel therapy in myelofibrosis, graft-versus-host disease, and HLH. Its role in these diseases is thought to extinguish inflammatory factor storms by inhibiting the JAK-STAT pathway (Maschalidi et al., 2016). In a precious report, we have already successfully treated one CAEBV patient with ruxolitinib with long time survival in our center (Jin et al., 2019). Also, for refractory/relapse HLH patients, ruxolitinib has shown impressive effects on controlling inflammation-related clinical manifestations (fever, elevated sCD25 and ferritin, and elevated cytokines) (Wang et al., 2019). Considering that CAEBV has an inflammatory aspect, as hypercytokinemia is a common feature, we conducted this study to evaluate the therapeutic effect and adverse effects of ruxolitinib in CAEBV patients.

## Methods

### Patients

All patients who suffered active CAEBV and were treated with ruxolitinib as compassionate use in our center from Sep 1, 2017, and Apr 30, 2019, were retrospectively analyzed. The diagnostic criteria for CAEBV as defined in the recently revised World Health Organization classification include persistent IM-like symptoms for more than 3 months, increased EBV-DNA in peripheral blood, histological evidence of organ disease, and EBV-RNA or viral protein in affected tissues (Quintanilla-Martinez et al., 2017). Lymphoma was excluded by repeated pathological biopsy of the focal area and bone marrow biopsy.

Patients who have used CAEBV targeted therapy before (including antiviral regimen, corticosteroids, and chemotherapy), but with poor efficacy or with recurrence of CAEBV active symptoms, and patients who cannot undergo allo-HSCT immediately due to various reasons (such as financial considerations, hesitation about allo-HSCT, serious complications, and lack of donors) are included.

All patients included provided written informed consent to receive treatment with ruxolitinib and for blood sample collection.

### Treatment and Evaluation

Patients received ruxolitinib as a monotherapy. Refer to the previously reported dosage of ruxolitinib in relapsed/refractory HLH (Wang et al., 2019); the dose for patients (age ≥ 14 years) is generally 10 mg twice daily. For children (age < 14 years), the dose was generally 5 mg twice daily.

Clinical and laboratory evaluations were performed before therapy and every two weeks during therapy. There have been no standard effects evaluation criteria in the treatment of CAEBV. The main clinical manifestation of CAEBV, including temperature, size of spleen and liver, levels of ALT and AST, organ infiltration (EBV-DNA or EBV coding small RNA (EBER) detected in tissue), and PBMC EBV-DNA copies, was mainly performed and evaluated.

EBV-DNA copies detection: PBMC, plasma, and other liquid specimens were amplified by real-time fluorescent quantitative PCR (qPCR) and TaqMan hydrolysis probes. The EBV-specific DNA fragments in the hydrolyzed samples were amplified, and the EBV-DNA in the samples was quantified according to the standard curve established by the standard. Pass the EBV international standard, namely, 09/260 (NIBSC number) for detection. Intracellular EBV-DNA copies were quantified by qPCR in sorted B-, T-, and NK-cells.

### Adverse Effects

The main side effects of ruxolitinib are the possibility of leukopenia, thrombocytopenia, elevated transaminases, elevated bilirubin, elevated triglycerides, pneumonia, and urinary tract infection. Related indicators are monitored to evaluate the AE situation.

### Outcome

The continuing remission period was calculated from the usage of ruxolitinib to the discontinuation of it. For patients who are still using ruxolitinib as of the analysis time (Oct 31, 2020), the period is calculated from the beginning of using ruxolitinib to the analysis time. The main reasons for discontinuing ruxolitinib include disease recurrence, turning to allo-HSCT, patient’s death, change of treatment plan, and long-term remission.

### Statistical Analysis

SPSS 22.0 (IBM, New York/United States) statistical software was adopted, and data that did not fit a normal distribution are presented as median and range. *t*-test was used for data that fit a normal distribution and homogeneity of variance, and the Wilcoxon rank-sum test was used for others. *p* < 0.05 was considered to denote a significant difference.

## Results

### Patients and Clinical Manifestation

A total of nine patients (median age, 16 years; range, 6–28 years), including six males and three females, with symptomatic CAEBV were enrolled (Table 1). Before ruxolitinib, all patients were presenting with typical active CAEBV signs, including fever (6/9), liver function abnormalities (4/9), hepatosplenomegaly (7/9), lymphadenopathy (6/9), lesions on scans (two with skin infiltration and one with subcutaneous nodules, liver, and bone marrow), and cytopenia (2/9). All of these patients suffered elevated EBV-DNA copies in PBMC (median 5.2*10^3^ copies/ml, range 5.5*10^2^–1.2*10^6^ copies/ml). Seven patients detected intracellular EBV-DNA copies in the sorted B-, T-, and NK-cells, and they all detected EBV-DNA on T-, NK-, and B-cells.

**TABLE 1:** Clinical and laboratory manifestation before ruxolitinib treatment.

Case	Gender	Age (years)	Symptoms	WBC (*10^9^/L)	PLT (*10^9^/L)	AST (U/L)	ALT (U/L)	EBV-DNA (PBMC, copies/ml)	EBV-DNA copies in sorted B-cells (CD19^+^)	EBV-DNA copies in sorted CD4+T-cells	EBV-DNA copies in sorted CD8+T-cells	EBV-DNA copies in sorted NK-cells (CD56^+^)	Organ infiltration (EBER+)
1	M	20	Fever and splenomegaly	3.81	162	21.6	38	2.30*10^3^	7.7*10^5^	9.4*10^6^	3.2*10^6^	5.7*10^3^	
2	M	16	Fever, splenomegaly, and cytopenia	2.17	74	32.9	32	5.50*10^2^	2.4*10^4^	1.9*10^4^	6.5*10^4^	5.9*10^4^	Skin
3	F	28	Splenomegaly, liver function abnormalities, and lymphadenopathy	3.58	194	45.3	43	1.30*10^3^	8.9*10^3^	2.7*10^3^	2.9*10^3^	9.6*10^4^	Subcutaneous nodules, liver, and bone marrow
4	F	23	Fever, splenomegaly, and liver function abnormalities	6.89	187	59.1	69	1.10*10^5^	1.0*10^7^	2.2*10^5^	6.2*10^6^	1.3*10^8^	—
5	M	13	Lymphadenopathy	6.72	173	11.6	9	1.30*10^3^	—	—	—	—	—
6	M	7	Fever, splenomegaly, liver function abnormalities, and lymphadenopathy	12.52	311	175.4	243	1.20*10^6^	3.2*10^6^	0	3.0*10^6^	2.4*10^7^	Skin
7	M	19	Splenomegaly and lymphadenopathy	4.89	240	36.5	23	5.70*10^4^	7.7e*10^5^	1.9*10^6^	1.4*10^6^	1.4*10^6^	—
8	M	9	Fever, splenomegaly, and cytopenia	2.69	105	36	35	5.20*10^3^	—	—	—	—	—
9	F	6	Fever, liver function abnormalities, and lymphadenopathy	8.11	347	58.3	41	1.40*10^4^	5.7*10^3^	4.0*10^2^	3.3*10^3^	1.6*10^4^	—

Eight patients had received CAEBV targeted treatment before ruxolitinib (five patients of corticosteroids/IVIG, two patients of chemotherapy, and one patient of Pegaspargase). The median time from the diagnosis of CAEBV to the use of ruxolitinib was four months (range 0.3–39.0 months). Five patients were poorly controlled with previous therapy, and three patients experienced recurrence of active CAEBV symptoms.

### Treatment and Outcome

**FIGURE 1:**
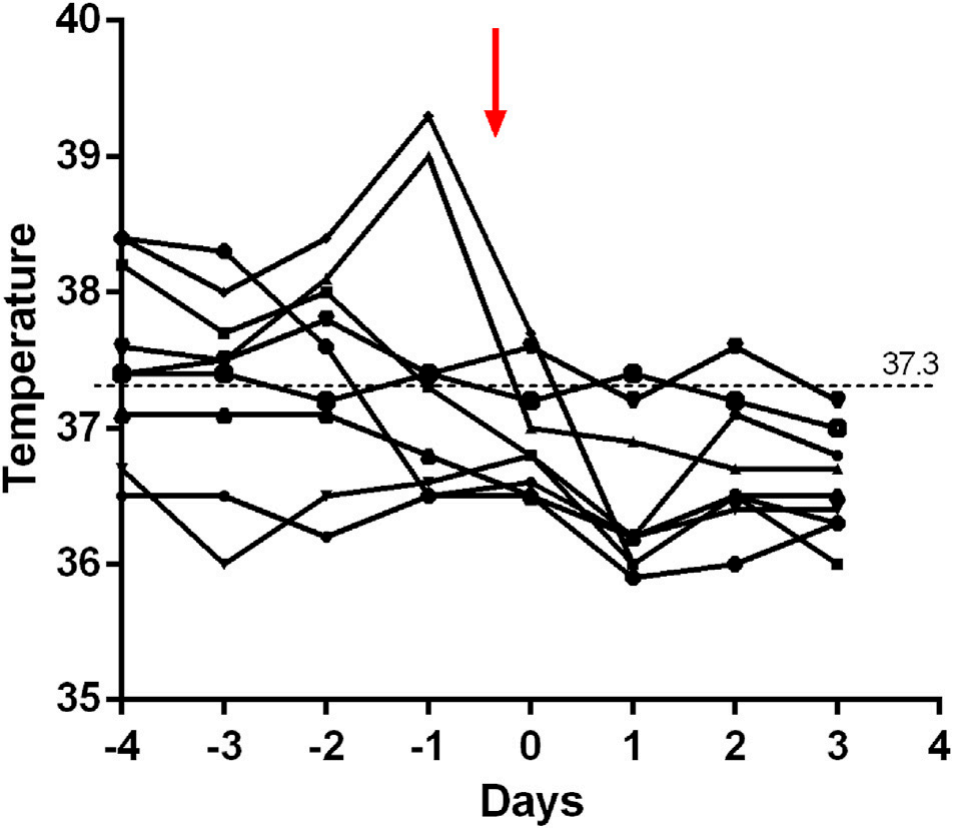
Fever extinguished in patients treated with ruxolitinib within 48 h. Red arrow, ruxolitinib intervention.

**FIGURE 2:**
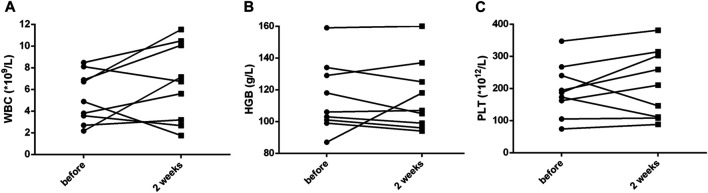
Cytopenia recovering after treatment with ruxolitinib. **(A)** White blood cell count before and after ruxolitinib treatment. **(B)** Hemoglobin concentration before and after ruxolitinib treatment. **(C)** Platelet count before and after ruxolitinib treatment. WBC, white blood cells; HGB, hemoglobin; PLT, platelets.

**FIGURE 3:**
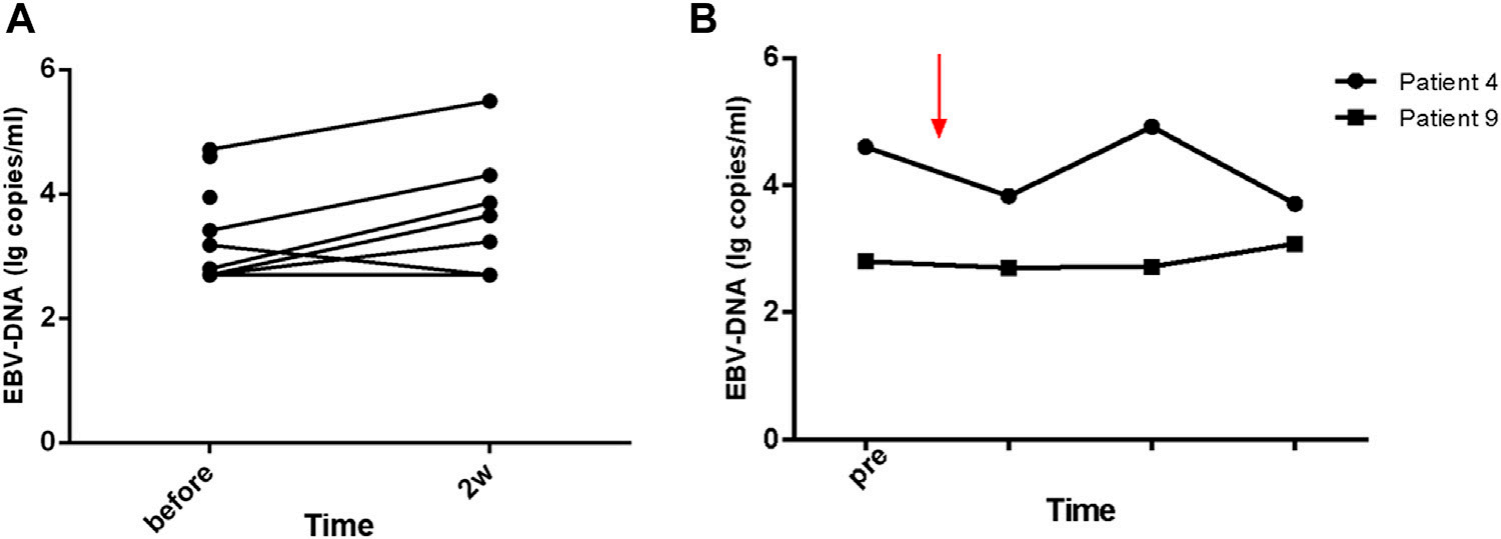
The change of EBV-DNA copy number with the treatment of ruxolitinib. **(A)** EBV-DNA persists in the peripheral blood of patients treated with ruxolitinib. **(B)** The kinetics of EBV-DNA copy number in patients with long-term stable remission (patients 4 and 9) before and during ruxolitinib monotherapy.

Within 48 h of starting ruxolitinib treatment, five out of six patients suffering fever became afebrile (Figure 1). Among the seven patients with splenomegaly, two patients had their splenomegaly returned to normal after ruxolitinib treatment. The AST/ALT level of three out of four patients decreased after ruxolitinib treatment, but none of them achieved normal levels. Two patients with cytopenia recovered after ruxolitinib treatment (Figures 2A–C). In general, seven out of nine patients responded to ruxolitinib (patients 1, 2, 3, 4, 5, 6, 8, and 9). No significant decrease in EBV-DNA copy number was observed after ruxolitinib (*p* = 0.161) (Figure 3A).

**FIGURE 4:**
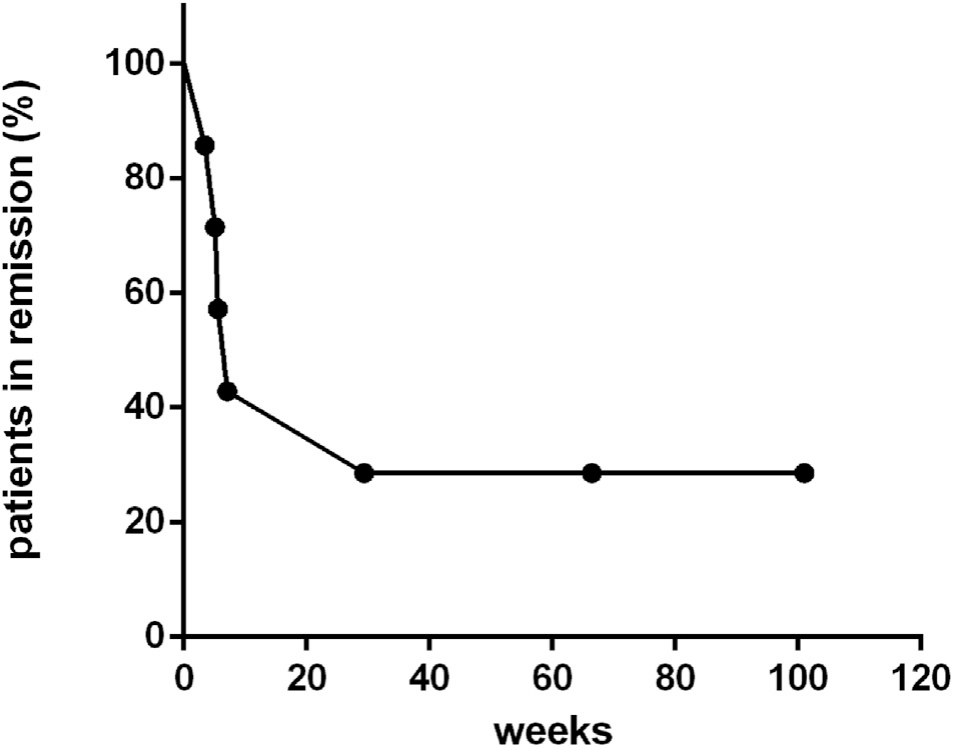
Continuing period in remission of patients treated with ruxolitinib.

For those seven patients who responded to ruxolitinib, the median continuing period in remission was 7.1 weeks (range, 3.4–123.7 weeks) (Figure 4). Two patients achieved long-term stable remission (considered as continuous remission for at least 48 weeks without any recurrence) with ruxolitinib monotherapy (patients 4 and 9). Both of them had stopped the treatment of ruxolitinib as of the follow-up time (Oct 31, 2020). The kinetics of EBV-DNA copy number before and during ruxolitinib monotherapy of patients 4 and 9 are presented in Figure 3B. Even though the peripheral blood of patients 4 and 9 still had low copies of EBV-DNA, there were no signs of active CAEBV until follow-up. Two patients were bridged to allo-HSCT successfully (patients 5 and 6). Three patients suffered a relapse and stopped ruxolitinib treatment (patients 1, 2, and 7). Patient 1 turned to chemotherapy and cell therapy (EBV-CTL). Patient 2 converted to chemotherapy. After eight courses, he was unable to tolerate allo-HSCT due to severe pneumonia and finally died. Patient 7 was unable to undergo allo-HSCT due to congenital heart disease. He relapsed after reducing the dose of ruxolitinib and was complicated with HLH. The HLH-directed treatment was not effective and he eventually died. The other two patients who did not respond to ruxolitinib stopped ruxolitinib and switched to other treatment plans. The details of treatment-related information were summarized in Table 2.

**TABLE 2:** Treatment details and response.

Case	Previous therapy	Duration from diagnosis to ruxolitinib (months)	Dosage of ruxolitinib	Response	Continuing period in remission (weeks)	Outcome	Survival
1	Pegaspargase	4.00	10 mg twice daily	Y	7.1	Relapse→chemotherapy + EBV-CTL	Survival
2	Chemotherapy	37.03	10 mg twice daily	Y	5.1	Relapse→chemotherapy	Death
3	Corticosteroids	33.53	10 mg twice daily	N	—	—	—
4	Chemotherapy	39.00	10 mg twice daily	Y	101.0	Long-term remission	Survival
5	Corticosteroids	0.27	10 mg twice daily	Y	5.6	Allo-HSCT	Survival
6	N/A	19.87	5 mg twice daily	Y	3.4	Allo-HSCT	Survival
7	Corticosteroids	0.13	10 mg twice daily	Y	29.4	Relapse→chemotherapy	Death
8	Corticosteroids	0.83	5 mg twice daily	N	—	—	—
9	Corticosteroids	1.33	5 mg twice daily	Y	89.7	Long-term remission	Survival

### Safety

Transient leukopenia was detected in two patients (grades 2 and 3, CTCAE 5.0). Two patients suffered transaminases (grades 2 and 3, CTCAE 5.0), one of them was considered to be drug-related (non-ruxolitinib-related) liver damage, and the other was considered to be caused by CAEBV liver infiltration. No thrombocytopenia, hypertriglyceridemia, or urinary infection has been observed.

## Discussion

CAEBV is now regarded as a prototype of EBV-associated T- or NK-cell lymphoproliferative diseases (EBV + T/NK-LPDs) in the 2016 WHO classification (Quintanilla-Martinez et al., 2017). In certain circumstances, CAEBV will develop hemophagocytic lymphohistiocytosis (HLH), lymphoma, or multiorgan failure, which may lead to rapid progression and finally death (Kimura et al., 2001; Kimura et al., 2012). Even with a mild and self-limiting clinical manifestation, the prognosis of CAEBV is very poor. Three-year OS in patients with uncontrolled active disease is only 16.7% (Sawada et al., 2017). In a prospective analysis of 108 EBV-LPD cases, 44% of the patients died with a 46-month median follow-up period (Kimura et al., 2012). The only effective treatment strategy for a cure currently is allo-HSCT (Arai, 2019). Almost all of the current therapy, for CAEBV, except for allo-HSCT, is unsatisfactory and at best transiently delays the progression of the disease, including antiviral therapy, immunomodulatory agents, and corticosteroids. The three-step therapy proposed by Kawa et al. only provides better suppressed disease activities to bridge to allo-HSCT (Sawada et al., 2017). Considering that even though CAEBV was regarded as a kind of EBV-LPD disease, it still has an inflammatory aspect, as hypercytokinemia is a common feature (Arai, 2018; Kimura, 2018; Onozawa et al., 2018). And, in a precious report, we have already successfully treated one CAEBV patient with ruxolitinib with long-term survival in our center (Jin et al., 2019). Here, this study, to our acknowledge, is the first retrospective series of patients using ruxolitinib in CAEBV.

The overproduction of T-cell-derived cytokines, including interferon-γ (IFN-γ), and the phosphorylation-dependent activation of the Janus family kinases JAK1 and JAK2 are hallmarks of the final common pathway in inflammatory (Ohga et al., 2001; Aaronson, et al., 2002).

Ruxolitinib, the JAK1/2 inhibitor, is currently observed to be effective in the HLH mouse model (Maschalidi et al., 2016; Albeituni et al., 2019), whose role in HLH is thought to extinguish inflammatory factor storms by inhibiting the JAK-STAT pathway. Anecdotal clinical experience with ruxolitinib in HLH has been reported afterward (Broglie et al., 2017; Slostad et al., 2018; Zandvakili et al., 2018; Sin and Zangardi, 2019). In the clinical study reported by Jingshi Wang et al. (Wang et al., 2019), ruxolitinib shows efficacy when used as relapse/refractory HLH salvage therapy. In the open-label, single-center, pilot study of ruxolitinib in adult secondary HLH, it showed impressive safety and activity. In our study of CAEBV, we found out that ruxolitinib has a significant effect on controlling the body temperature of patients. Almost all fever was sustained within 48 h after ruxolitinib. It is worth noting that most of the patients in this study were patients who had failed previous treatment or had recurrence of CAEBV activity (refractory/relapse). In this case, ruxolitinib can still effectively control fever. Ruxolitinib also plays a role in reducing liver damage and helping hematologic recovery, which is considered to be caused by inflammation. However, ruxolitinib’s effect in shrinking the enlarged spleen and reducing the number of EBV copies is not satisfactory. This is consistent with the previously published results of using ruxolitinib to treat relapsed and refractory HLH, which clarified that the role of ruxolitinib is mainly focused on temperature control, reducing ferritin, sCD25, and some fatal cytokines, but does not improve the status of EBV infection. These results suggest that the effect of ruxolitinib in CAEBV is mainly concentrated on its inflammation aspect, but not on its tumor-like characteristics.

According to a previous report, ruxolitinib was considered to be able to inhibit JAK activity by competitively inhibiting the ATP binding site of JAK kinase and may not only suppress the production of inflammatory cytokines of CAEBV patients but also decrease the viable cell number of EBV-positive NK or T cell lines and PBMCs by suppressing the phosphorylation of STAT3 in cells (Kimura, 2018; Onozawa et al., 2018). However, in the observation of this study, it seems that the main role of ruxolitinib is concentrated in inhibiting the excessive inflammatory but is not able to reduce the number of living cells of EBV-positive cells. Even in the two patients with long-term remission, there is still a copy number of EBV-DNA in PBMC. The effect of clearing EBV infection cannot be achieved. In fact, in this study, the remission continuing period of CAEBV patients with ruxolitinib was also not long enough (median 7.1 weeks). EBV clearance remains the only way to improve prognosis after disease stabilization, and allo-HSCT still seems to be the only way to finally cure. But when it comes to creating the conditions for allo-HSCT, ruxolitinib provides a good option. The outcomes of allo-HSCT for CAEBV patients with an active disease were significantly poorer than those with inactive disease (Kawa et al., 2011; Arai et al., 2016). Ruxolitinib seems to be an ideal bridge between CAEBV and allo-HSCT, taking into account other current treatments, such as the serious side effects of chemotherapy, opportunistic infections with corticosteroids, and the poor effects of antiviral treatment. Besides, controlling the activity of CAEBV can not only improve the quality of life of patients but also greatly help reduce the multiorgan involvement of CAEBV and reduce the acute progression of CAEBV into the life-threatening severe cytokine storm state, HLH.

Ruxolitinib was well tolerated and with no severe adverse effects. Thrombocytopenia, the most common side effect of ruxolitinib (Ahmed et al., 2019), was not common in this study. Ruxolitinib was not discontinued because of any adverse effects and the above adverse effects were alleviated, while ruxolitinib was continuously taken. This study still has certain limitations. Retrospective studies may cause bias in the results, the number of cases is rather small, and there is a lack of research on the effective mechanism of ruxolitinib in CAEBV. In the future, large-scale, prospective, randomized controlled studies are still needed to clarify the actual role of ruxolitinib in CAEBV.

## Conclusion

This is the first retrospective series of patients using ruxolitinib for the treatment of CAEBV. Ruxolitinib is effective in the treatment of active CAEBV in controlling body temperature and alleviating liver injury, those kinds of inflammatory status’s aspects. However, ruxolitinib did not show an ability to eliminate EBV-DNA. In general, ruxolitinib is an effective and rather safe option for controlling the symptoms of active CAEBV, especially in patients with CAEBV who have failed previous treatments or have relapsed. It can also play a promising role in improving the quality of daily life of patients and successfully bridging to allo-HSCT.

## Data Availability

The original contributions presented in the study are included in the article/supplementary material; further inquiries can be directed to the corresponding author.

## References

[B1] Aaronson D. S. Horvath C. M. (2002). A Road Map for Those Who Don't Know JAK-STAT. Science 296 (5573), 1653–1655. 10.1126/science.1071545 12040185

[B2] Ahmed A. Merrill S. A. Alsawah F. Bockenstedt P. Campagnaro E. Devata S. (2019). Ruxolitinib in Adult Patients with Secondary Haemophagocytic Lymphohistiocytosis: an Open-Label, single-centre, Pilot Trial. Lancet Haematol. 6 (12), e630–e637. 10.1016/s2352-3026(19)30156-5 31537486 PMC8054981

[B3] Albeituni S. Verbist K. C. Tedrick P. E. Tillman H. Picarsic J. Bassett R. (2019). Mechanisms of Action of Ruxolitinib in Murine Models of Hemophagocytic Lymphohistiocytosis. Blood 134 (2), 147–159. 10.1182/blood.2019000761 31015190 PMC6624972

[B4] Arai A. (2019). Advances in the Study of Chronic Active Epstein-Barr Virus Infection: Clinical Features under the 2016 WHO Classification and Mechanisms of Development. Front. Pediatr. 7, 14. 10.3389/fped.2019.00014 30805320 PMC6370717

[B5] Arai A. (2018). Chronic Active Epstein-Barr Virus Infection: a Bi-faceted Disease with Inflammatory and Neoplastic Elements. Immunol. Med. 41 (4), 162–169. 10.1080/25785826.2018.1556030 30704352

[B6] Arai A. Sakashita C. Hirose C. Imadome K.-I. Yamamoto M. Jinta M. (2016). Hematopoietic Stem Cell Transplantation for Adults with EBV-Positive T- or NK-Cell Lymphoproliferative Disorders: Efficacy and Predictive Markers. Bone Marrow Transpl. 51 (6), 879–882. 10.1038/bmt.2016.3 26901705

[B7] Bollard C. M. Cohen J. I. (2018). How I Treat T-Cell Chronic Active Epstein-Barr Virus Disease. Blood 131 (26), 2899–2905. 10.1182/blood-2018-03-785931 29712633 PMC6024635

[B8] Broglie L. Pommert L. Rao S. Thakar M. Phelan R. Margolis D. (2017). Ruxolitinib for Treatment of Refractory Hemophagocytic Lymphohistiocytosis. Blood Adv. 1 (19), 1533–1536. 10.1182/bloodadvances.2017007526 29296794 PMC5728466

[B9] Cohen J. I. Jaffe E. S. Dale J. K. Pittaluga S. Heslop H. E. Rooney C. M. (2011). Characterization and Treatment of Chronic Active Epstein-Barr Virus Disease: a 28-year Experience in the United States. Blood 117 (22), 5835–5849. 10.1182/blood-2010-11-316745 21454450 PMC3112034

[B10] Jin Z. Wang Y. Wang J. Zhang J. Wu L. Wang Z. (2019). Long-term Survival Benefit of Ruxolitinib in a Patient with Relapsed Refractory Chronic Active Epstein-Barr Virus. Ann. Hematol. 98 (8), 2003–2004. 10.1007/s00277-019-03647-5 30830248 PMC6647074

[B11] Kawa K. K. Sawada M. Sato N. Okamura T. Sakata S. Kondo M. (2011). Excellent Outcome of Allogeneic Hematopoietic SCT with Reduced-Intensity Conditioning for the Treatment of Chronic Active EBV Infection. Bone Marrow Transpl. 46 (1), 77–83. 10.1038/bmt.2010.122 20498651

[B12] Kimura H. Hoshino Y. Kanegane H. Tsuge I. Okamura T. Kawa K. (2001). Clinical and Virologic Characteristics of Chronic Active Epstein-Barr Virus Infection. Blood 98 (2), 280–286. 10.1182/blood.v98.2.280 11435294

[B13] Kimura H. Ito Y. Kawabe S. Gotoh K. Takahashi Y. Kojima S. (2012). EBV-associated T/NK-cell Lymphoproliferative Diseases in Nonimmunocompromised Hosts: Prospective Analysis of 108 Cases. Blood 119 (3), 673–686. 10.1182/blood-2011-10-381921 22096243

[B14] Kimura H. (2018). JAK Inhibitors for Refractory Lymphoma. Oncotarget 9 (68), 32883–32884. 10.18632/oncotarget.26054 30250636 PMC6152482

[B15] Maschalidi S. Sepulveda F. E. Garrigue A. Fischer A. de Saint Basile G. (2016). Therapeutic Effect of JAK1/2 Blockade on the Manifestations of Hemophagocytic Lymphohistiocytosis in Mice. Blood 128 (1), 60–71. 10.1182/blood-2016-02-700013 27222478

[B16] Ohga S. Nomura A. Takada H. Ihara K. Kawakami K. Yanai F. (2001). Epstein‐Barr Virus (EBV) Load and Cytokine Gene Expression in Activated T Cells of Chronic Active EBV Infection. J. Infect. Dis. 183 (1), 1–7. 10.1086/317653 11106535

[B17] Okano M. Kawa K. Kimura H. Yachie A. Wakiguchi H. Maeda A. (2005). Proposed Guidelines for Diagnosing Chronic Active Epstein-Barr Virus Infection. Am. J. Hematol. 80 (1), 64–69. 10.1002/ajh.20398 16138335

[B18] Olson G. Kanaan M. Gersuk G. Kelley L. Jones J. (1986). Correlation between Allergy and Persistent Epstein-Barr Virus Infections in Chronic-Active Epstein-Barr Virus-Infected Patients. J. Allergy Clin. Immunol. 78 (2), 308–314. 10.1016/s0091-6749(86)80081-1 3016066

[B19] Onozawa E. Shibayama H. Takada H. Imadome K.-I. Aoki S. Yoshimori M. (2018). STAT3 Is Constitutively Activated in Chronic Active Epstein-Barr Virus Infection and Can Be a Therapeutic Target. Oncotarget 9 (57), 31077–31089. 10.18632/oncotarget.25780 30123428 PMC6089567

[B20] Quintanilla-Martinez L. K. Y. Kimura H. Jaffe E. S. (2017). “EBV-positive T-Cell and NK-Cell Lymphoproliferative Diseases of Childhood,” in WHO Classification of Tumours of Haematopoietic and Lymphoid Tissue. Editors Swerdlow S. Campo E. Harris N. L. (Lyon IARC Press), 355–363.

[B21] Quintanilla-Martinez L. Kumar S. Fend F. Reyes E. Teruya-Feldstein J. Kingma D. W. (2000). Fulminant EBV+ T-Cell Lymphoproliferative Disorder Following Acute/chronic EBV Infection: a Distinct Clinicopathologic Syndrome. Blood 96 (2), 443–451. 10.1182/blood.v96.2.443 10887104

[B22] Sawada A. Inoue M. Kawa K. (2017). How We Treat Chronic Active Epstein-Barr Virus Infection. Int. J. Hematol. 105 (4), 406–418. 10.1007/s12185-017-2192-6 28210942

[B23] Sin J. H. Zangardi M. L. (2019). Ruxolitinib for Secondary Hemophagocytic Lymphohistiocytosis: First Case Report. Hematol.Oncol. Stem Cel. Ther. 12 (3), 166–170. 10.1016/j.hemonc.2017.07.002 28834694

[B24] Slostad J. Hoversten P. Haddox C. L. Cisak K. Paludo J. Tefferi A. (2018). Ruxolitinib as First-Line Treatment in Secondary Hemophagocytic Lymphohistiocytosis: A Single Patient Experience. Am. J. Hematol. 93 (2), E47–E49. 10.1002/ajh.24971 29134683

[B25] Wang J. Wang Y. Wu L. Wang X. Jin Z. Gao Z. (2019). Ruxolitinib for Refractory/relapsed Hemophagocytic Lymphohistiocytosis. Haematologica 105, e210. 10.3324/haematol.2019.222471 31515353 PMC7193462

[B26] Zandvakili I. Conboy C. B. Ayed A. O. Cathcart-Rake E. J. Tefferi A. (2018). Ruxolitinib as First-Line Treatment in Secondary Hemophagocytic Lymphohistiocytosis: A Second Experience. Am. J. Hematol. 93 (5), E123–E125. 10.1002/ajh.25063 29417621

